# *In vitro* culture and confocal microscopy study of *Maritrema gratiosum* Nicoll, 1907 (Digenea): From metacercaria to ovigerous adult

**DOI:** 10.1007/s00436-024-08446-0

**Published:** 2025-01-09

**Authors:** YuChing Chuang, Andrew P. Shinn, James E. Bron

**Affiliations:** 1 Institute of Aquaculture, University of Stirling, 5F.-2, No. 196, Sec. 2, Xinglong Rd., Wenshan Dist., Taipei City, 116096 Taiwan (R.O.C.); 2 INVE (Thailand) Limited, Nonthaburi, 11120 Thailand; 3 Institute of Aquaculture, University of Stirling, Stirling, Scotland FK9 4LA UK

**Keywords:** Metacercarial development, Barnacle parasites, Excystment rates, Egg production, Spermatogenesis, Musculature

## Abstract

This study set out to characterise the *in vitro* development, including musculature, of the microphallid parasite of the barnacle *Semibalanus balanoides* (Linnaeus, 1767), *Maritrema gratiosum* Nicoll, 1907 collected in Scotland*.* An *in vitro* culture model was developed to obtain ovigerous adults of *M. gratiosum* and their morphology was observed. Different media were tested and NCTC-109 was chosen as the best medium. The effects of different concentrations of serum upon adult longevity, size and egg production was measured. Survival for 10-days was achieved when flukes were cultured in NCTC-109 plus chicken serum and antibiotics. Forty percent chicken serum seemed to provide better results in terms of survival time and producing flukes with the largest body lengths. Both normal and abnormal eggs were observed from adults cultured *in vitro*. Confocal microscopy was undertaken to provide details of the development of the parasite’s ultrastructure, including musculature, during the course of *in vitro* culture. While the musculature of *M. gratiosum* was similar to that of other microphallids, some additional novel structures were observed, most notably a ligament connecting *pars prostatica* and seminal vesicle and a racket-shaped excretory bladder. This study has provided greater insight into the biology *M. gratiosum*, and also developed a good *in vitro* model which might be applied to ecological or medical research in the future.

## Introduction

*In vitro* culture of digenean life-cycles is challenging due to the complexity of multiple life stages and diverse host requirements. Each life stage of Digenea has different cultivation needs, influenced by their hosts, which range across various phyla like Mollusca and Chordata. Factors such as nutritional needs, temperature, oxygen levels, and osmolality vary accordingly. Additionally, growth requirements can differ significantly even among species within the same genus. *Maritrema gratiosum* Nicoll, 1907 (syn. *M. arenaria*) is a microphallid digenean with a life cycle involving the periwinkle as the first intermediate host, the barnacle *Semibalanus balanoides* (Linnaeus, 1767) as the second intermediate host, and various wading birds as the final hosts (Deblock 2008). This study focuses specifically on this distome digenean.

Currently, individual or several life stages of digenean species can be cultured in the lab using axenic or synxenic methods, but this is often limited to short durations (Ractliffe et al. 1969; Davies and Smyth 1978; Basch 1981a, b; Yoshino and Laursen 1995; Augot et al. 1997; Loker et al. 1999; Ivanchenko et al. 1999; McCusker et al. 2016). Excystment, the first step in culturing metacercariae *in vitro*, has been widely studied, with methods varying across species (Lackie 1975; Smyth and Halton 1983; Fried 1994). Some species excyst easily under certain temperature conditions, while others require enzymatic treatments. For instance, *M. gratiosum* can excyst using a specific solution (Irwin 1983), but other species like *Microphallus abortivus* Deblock, 1974 require more complex treatments (Davies and Smyth 1979; Saville and Irwin 1991).

The culture of progenetic digenean species, which mature earlier, is easier than that of non-progenetic species like *Fasciola hepatica* Linnaeus, 1758 and *Schistosoma mansoni* Sambon, 1907, which are important in veterinary and medical research. For progenetic species, simple temperature increases can stimulate spermatogenesis, oogenesis and vitellogenesis (Smyth and Halton 1983). For example, excysted metacercariae of *Microphallus similis* (Jägerskiöld, 1900) Baer, 1944, developed into ovigerous adults after being cultured in specific saline solutions (Davies and Smyth 1978). In another case, *Microphallus turgidus* (Leigh, 1958) was successfully cultured *in vitro* to produce fertile eggs that could infect snail hosts (Pung et al. 2011).

Studies have shown that culture media such as Hanks’ saline, RPMI-1640, and DME/F-12 can support the development of these parasites, although the fertility of the produced eggs can vary. *Maritrema gratiosum* was first successfully cultured *in vitro* by Zaben (1988), who optimized conditions to support survival and egg production, with the best results achieved in medium 199 supplemented with foetal bovine serum (FBS).

Understanding the muscular systems of digeneans is crucial for studying their biology and development. The muscular systems of parasitic flatworms are generally similar, consisting of somatic muscles, adhesive organ muscles, and muscles of the alimentary and reproductive systems. To observe muscular systems, a fluorescent phalloidin stain, which binds to muscle F-actin, is applied in conjunction with fluorescent light microscopy, particularly laser scanning confocal microscopy (LSCM) to provide a powerful method to reveal the structures of flatworm muscular systems. In digenean studies, this technique is often applied in combination with immunofluorescent techniques to reveal the neuromusculature or is used alone for the study of muscular systems. It has been applied to adult stages (Mair et al. 1998; 2000), to metacercarial stages (Stewart et al. 2003a), and to follow changes to morphology throughout parasite development (Stewart et al. 2003b; Šebelová et al. 2004; Bulantová et al. 2011; Petrov and Podvyaznaya 2016; Borges et al. 2017). Very few studies have been conducted concerning the musculature of microphallids. Krupenko and Dobrovolski (2018) stated that the whole ventral surface of microphallid parasites (e.g., the metacercariae of *Microphallus piriformes* Galaktionov, 1983 and *Microphallus pygmaeus* (Levinsen, 1881) Baer, 1944, and the adult of *Levinseniella branchysoma* (Creplin, 1837) Stiles et Hassall, 1902) acts as an attachment organ (ventral concavity). In this study, a three-layer arrangement of somatic muscles—circular, longitudinal, and diagonal—was observed. Additional muscle sets in the hind body supported the shape and function of the ventral concavity. Thicker internal muscles near the ventral sucker were also noted. The authors suggested that these ventral muscles generate negative pressure for attachment, working with surface spines to strengthen the attachment of these small digeneans, which have relatively weak ventral suckers.

DAPI (4,6-diamidino-2-phenylindole), a fluorescent dye highlighting cell nuclei, has been used in limited studies to elucidate tissue structures in digeneans (Kremnev et al. 2021, 2023; Krupenko et al. 2023). In this study, DAPI staining is used to characterize the cellular structures of *M. gratiosum* throughout its development from metacercariae to ovigerous adults.

## Research objectives

The first objective of this study was to culture metacercariae of *M. gratiosum* under *in vitro* conditions. This involved the optimisation of culture conditions to promote spermatogenesis and self-fertilization and to obtain ovigerous adults with normal eggs to provide materials for a study of development. The development of *M. gratiosum* from excystment through to the adult stage has not been thoroughly studied using modern techniques and, furthermore, no study has been performed to examine the musculature of *M. gratiosum* during development. Therefore, by means of confocal microscopy, the second objective of this study was to investigate the muscular systems, reproductive organs, and tegumental glandular structures of *M. gratiosum* during development from metacercariae to ovigerous adults, and to deduce their biological function.

## Materials and methods

### Collection of barnacle samples and their maintenance

Samples of the barnacle *S. balanoides*, the second intermediate host of *M. gratiosum*, were collected from two locations in Dunbar, southeast Scotland: a rocky shore at "Red Rock" (56° 00′ 19.9"N; 2° 31′ 33.4"W) and "the Leisure Pool" (56° 00′ 19.5"N; 2° 31′ 04.2"W), below a kittiwake, *Rissa tridactyla* (Linnaeus, 1758), breeding cliff. Small rock pieces with barnacles attached were maintained in aerated aquaria with 35 ppt seawater at 14 °C ± 0.5 °C in a lab incubator. The water was changed weekly, with 80% of the total volume replaced, and the systems were maintained for two months. The barnacles were not provided with food during their maintenance in the laboratory.

### Parasite material collection

Barnacles were detached from the rock substrate using a scalpel and tweezers. The entire barnacle was placed in a glass with seawater, and the prosoma was extracted from the shell plates using tweezers. The prosoma was then examined under a coverslip to count the parasite cysts per barnacle. Afterward, the coverslip was removed, and the oral cone was cut with a scalpel. The prosoma, with the cysts inside, was partially torn to release the cysts, which were then placed in a 1.5 mL Eppendorf tube with 500 µL of 0.01 M phosphate-buffered saline (PBS). The Eppendorf tubes were incubated at 4 °C ± 0.5 °C overnight. This procedure was conducted for logistical reasons and to standardize the process, as there is typically not enough time to complete the experiment within a single day. However, the metacercariae can be used immediately after extraction from the host.

### *In vitro* culture experiment

#### Preparation of excystment fluid and culture media

PBS (P4417, Sigma-Aldrich, UK) was prepared at a concentration of 0.01 M. Two cell culture media, Eagle's Minimum Essential Medium (EMEM, Gibco, Paisley, Scotland) and NCTC 109 without glutamine (ThermoFisher Scientific, Oxford, UK), were evaluated for their ability to support parasite development. Chicken serum (CS, Sigma) and 20% fetal bovine serum (FBS, Gibco) were used as food sources in the NCTC 109 experiments. For the EMEM experiments, EMEM complete medium (with 2 mM L-glutamine), 10% FBS, and non-essential amino acids (1×) were used. Various antibiotic concentrations were tested to determine the minimum effective levels for bactericidal/bacteriostatic activity, using a penicillin/streptomycin solution (10,000 units/mL penicillin and 10 mg/mL streptomycin, sterile-filtered, Sigma-Aldrich, Merck, UK) as a disinfectant. Small-scale experiments tested different combinations of excystment fluid and culture medium (data not shown). After optimization, a standard excystment fluid and culture medium were used in larger-scale experiments to assess the effects of varying CS concentrations.

#### Calculations of excystment percentage

The excystment percentage of metacercariae incubated in 0.01 M PBS at 40 °C ± 0.5 °C was calculated. Eight barnacles, each containing 10–30 cysts, were selected, with the cysts from each individual barnacle placed in a separate Eppendorf tube as a single experimental unit. Excystment percentages were recorded for two experimental units at 15, 30, 60, and 120 min.

#### Observations of spermatogenesis

Individual cysts (24 replicates) isolated from collected barnacles were placed in separate Eppendorf tubes containing 200 µL of 0.01 M PBS. The metacercariae were excysted in a water bath at 40 °C ± 0.5 °C. Metacercariae were examined for the presence of spermatozoa under a light microscope (Olympus BX51) using phase contrast and differential interference contrast (DIC) microscopy at 15, 30, 60, and 120 min, with 6 individuals sampled and observed at each time point. Timing began from the moment each individual cyst was placed in the tube. As excystment times varied slightly between individuals, only those that had excysted by each time point were selected for observation.

#### Confirmation of self-fertilisation

To confirm self-fertilization, individual metacercariae were cultured in Eppendorf tubes with 200 µL of NCTC 109 supplemented with 20% CS, penicillin (100 U mL^−1^), and streptomycin (100 µg mL^−1^). Each metacercaria was excysted, transferred to the culture medium, and incubated at 40 °C ± 0.5 °C for 2 days under normal gas phase. After 4 days, all 30 flukes were examined for the presence of eggs using light microscopy (Olympus BX51) with phase contrast and DIC.

#### Assessing the performance of the *in vitro* culture method

A systematic experiment was conducted to evaluate the *in vitro* performance of excysted distomes of *M. gratiosum*. The effects of chicken serum on egg production and body growth of the excysted distomes in NCTC 109 culture medium were assessed. NCTC 109 was chosen based on pilot study results (data not shown). Four parameters—percentage survival, body length, body width, and egg number—were used to evaluate *in vitro* performance. A temperature of 40 °C ± 0.5 °C was applied for all the *in vitro* culturing experiments.

Two treatment groups were tested: Group 1 with NCTC 109 plus 20% CS, penicillin (100 U mL^−1^), and streptomycin (100 µg mL^−1^), and Group 2 with NCTC 109 plus 40% CS, penicillin (200 U mL^−1^), and streptomycin (200 µg mL^−1^). Metacercariae were extracted from barnacles, excysted in PBS for 4 h, and then allocated to tubes with 500 µL of culture medium. Culture media were changed every two days, and all procedures were carried out under non-sterile conditions due to the non-sterile source material.

Group 1 started with 26 tubes (130 excysted distomes, 5 flukes per tube), and Group 2 with 24 tubes (120 excysted distomes, 5 flukes per tube), based on parasite availability. The excysted distomes in each tube were checked daily for survival over a 10-day period. At 4, 24, 48, 72, 120, 168, and 216 h post-incubation in culture medium, two tubes from each group were observed, and growth indices were measured. Additionally, four tubes from Group 1 and six tubes from Group 2 were used for LSCM studies. Following measurement, the flukes were no longer viable for survival analysis and were therefore categorised as censored data.

Survival of the excysted distomes was defined by movement within 30 s or response to contact stimulation, along with a transparent body wall. Survival percentage was calculated by counting live flukes in each tube at each time point and dividing by the number of live flukes in each tube at time 0 (0 h, the time at which the metacercarial cysts were introduced into the culture medium). Body length and width of the surviving flukes were measured using Olympus cellSens software with a 10 × objective. Egg counts were performed under 20x/40 × objectives, and egg morphology was recorded. As described by Zaben (1988), normal eggs have an operculum and contain an ovum surrounded by vitelline globules. Morphological features, particularly in copulatory organs, were documented using light microscopy with or without phase contrast or DIC. Images of live flukes were captured under coverslip pressure to prevent movement.

Statistical analysis. Body length and width were compared using independent sample t-tests with a Bonferroni correction (adjusted significance *p* < 0.007) to account for multiple comparisons. Egg numbers were compared using the Mann–Whitney U test with Bonferroni correction (adjusted significance *p* < 0.008). Survival data (number of survivors) were presented in two formats: as a bar chart showing percentage survival relative to time 0, and through survival analysis using Kaplan–Meier curves, with differences assessed by the Log-Rank test (Mantel-Cox). All statistical analyses were conducted using IBM SPSS Statistics, versions 25 and 27.

### Laser scanning confocal microscopy (LSCM) observation

LSCM was used throughout the *in vitro* culture period to observe the development of body wall musculature, reproductive structures, and glandular structures in the tegument of the excysted distomes. Adult *M. gratiosum* samples were collected from the two treatment groups of the *in vitro* culture study. For the 20% CS group, two tubes were sampled at 72 and 120 h, with 5 flukes per tube. For the 40% CS group, samples were taken from two tubes at 48, 72, and 120 h. Excysted distomes were first transferred from culture medium to PBS, flattened under coverslip pressure, and fixed in 5% neutral buffered formalin (NBF) for 5 min in a humid chamber. The coverslip was then removed, and the specimens were washed back into PBS. The worms were stained with phalloidin (CytoPainter phalloidin-iFluor 594 Reagent, ABCAM) for 3 to 4 days in the dark at 4 °C. The staining solution was prepared with 30 µL of 2 × phalloidin conjugate, 0.2 µL of 10 mM DAPI, and 9 µL of 10% Triton ×100 in 900 µL of 5% NBF. After staining, the worms were mounted on slides, air-dried for about 10 min, and then covered with VECTASHIELD (Vector Laboratories) and sealed with nail varnish. The samples were observed using confocal microscopy (Leica TCS 2 AOBS LSCM) and images were captured with embedded software. In subsequent studies, freshly excysted or cultured samples were fixed in 4% paraformaldehyde or 10% NBF, either directly or after flattening under a coverslip. Staining procedures were adjusted to use 10× phalloidin instead of 8× or 2×. Flukes were washed in PBS with 0.1% Triton-X100 for 30 min before staining for approximately 3 days, followed by washing and mounting in VECTASHIELD.

## Results

### Calculation of excystment percentage

The excystment rates in 0.01 M PBS at 40 °C were 10.5% after 15 min, 66% after 30 min, 81% after 60 min, and 97.5% after 120 min (n = 149 total excysted metacercariae). Consequently, PBS was used as the standard excystment fluid in subsequent experiments unless specified otherwise.

### Observation of self-fertilisation and spermatogenesis

Of the 30 excysted distomes cultured individually, 26 contained eggs by day 4, with only 1 fluke dead and without eggs. Self-fertilisation was confirmed at 86%. Eggs from self-fertilisation were indistinguishable from those of cross-fertilisation, with no opercula present on either “normal” or “abnormal” eggs.

**Fig. 1:**
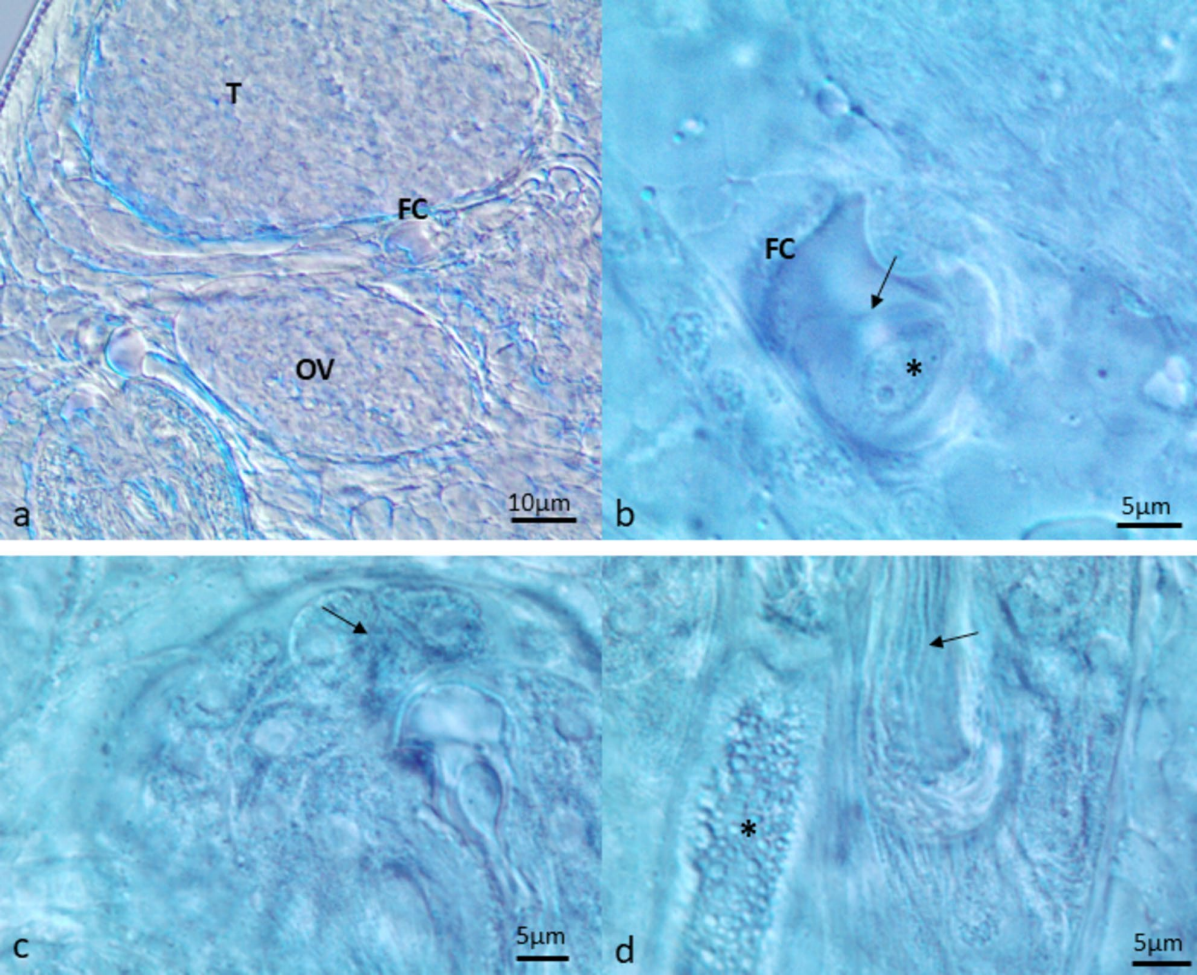
Fertilization process. **A** Note the refractive fertilization chamber between the testis and ovary (30 min in 0.01 M PBS). **B** Note a spermatozoan (arrow) and an ovum (asterisk) in the fertilization chamber (15 min in 0.01 M PBS). **C** Note the spermatozoa (arrow) in the seminal vesicle (60 min in 0.01 M PBS). **D** Spermatozoa in the duct (arrow) between the seminal vesicle and the *ductus ejaculatoris* (asterisk) (60 min in 0.01 M PBS). **FC**: fertilization chamber, **OV**: ovary, **T**: testis. Figures produced by Olympus cellSens software

Spermatogenesis was observed early in adult development. Spermatozoa were detected as soon as 15 min in the excysted flukes, in the fertilization chamber and *receptaculum seminis*, but not in the seminal vesicle. An ovum was also present in the fertilization chamber at this time. By 30 min, more spermatozoa were seen in the fertilization chamber (Fig. 1A, B). At 60 min, spermatozoa appeared in the seminal vesicles and *ductus ejaculatorius*, but the condition of the flukes was poor with a low sperm count (Fig. 1C, D). By 120 min, spermatozoa were again observed in the fertilization chamber but not in the seminal vesicle or *ductus ejaculatorius*.

### *In vitro* culture performance

Preliminary experiments revealed that (data not shown) flukes cultured in EMEM with FBS, L-glutamine, and NEAA exhibited abnormalities and high mortality, likely due to bacterial contamination. Conversely, flukes in PBS also exhibited abnormalities but survived until day three without egg production. NCTC-109, supplemented with antibiotics (100 U mL^−1^ penicillin and 100 µg mL^−1^ streptomycin) and serum, proved to be more successful. Flukes in NCTC-109 with 20% FBS maintained > 80% survival until 186 h, while those with 20% CS maintained > 60% survival until 192 h. These results align with Zaben (1988) findings, where cumulative mortality ranged from 40–51% by day 7–8 and reached 100*%* by day 13.

#### Survival analysis

**Fig. 2:**
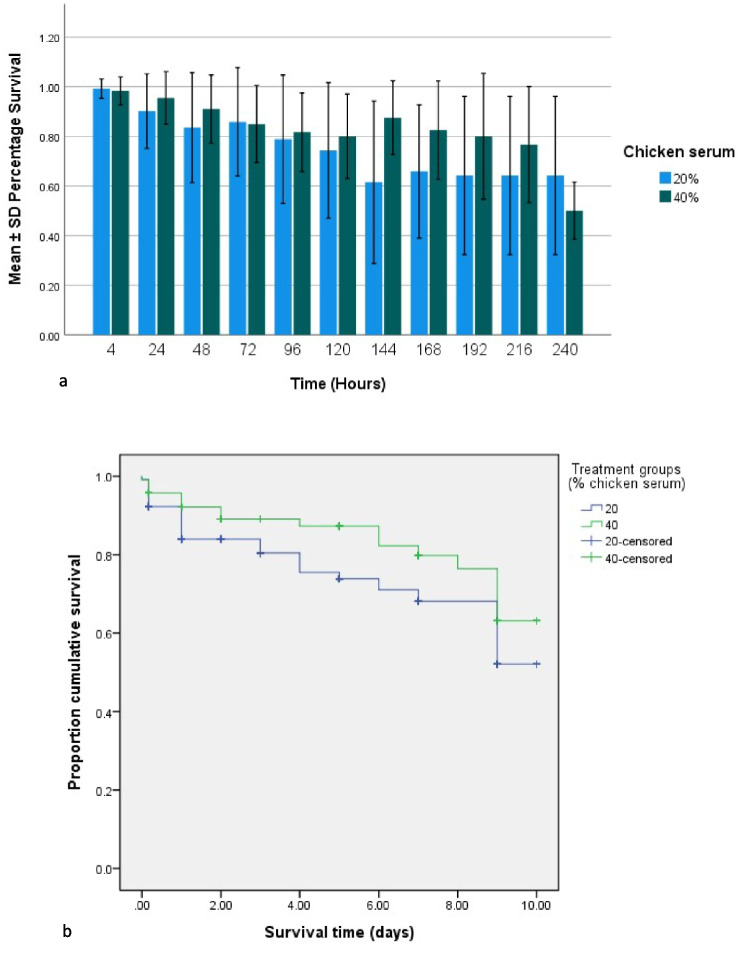
**A** Comparison of the mean ± SD percentage survival of excysted distomes of *Maritrema gratiosum* in NCTC with 20% (+ 100 U ml^-1^ penicillin + streptomycin 100 μg mL^-1^) and 40% chicken serum (+ 200 U ml^-1^ penicillin + streptomycin 200 μg mL^-1^) over 240 h post-excystment and 40ºC. **B** Survival curves for the two treatment groups according to the Kaplan–Meier method. Figures produced by IBM SPSS 25

In both treatment groups (20% and 40% CS), the number of survivors gradually declined, with cumulative survival remaining at or above 50% by day 10 (Fig. 2). Due to time and resource constraints, the experiment was concluded at 10 days. The mean survival time was 7.95 ± 4.29 days (Table 1), included here because the mean survival times for the two groups were quite similar, with additional details provided in Table 2. Although the Log-Rank test showed no significant difference between groups (*p* = 0.066), the 40% serum group exhibited higher survival rates at several time points, particularly in the later stages of the experiment (Fig. 2), with a slightly higher mean survival time of 8.43 ± 3.45 days (Table 1).

**Table 1:** Comparison of mean ± SE survival time of *Maritrema gratiosum* excysted distomes in 20% and 40% chicken serum + penicillin + streptomycin over 240 h and 40 °C

Serum	Mean Survival Time (days)			
	Estimate	Std. Error	95% Confidence Interval	
			Lower Bound	Upper Bound
20%	7.509	0.376	6.773	8.246
40%	8.431	0.316	7.812	9.050
Overall	7.948	0.250	7.458	8.437

**Table 2:** Comparison of growth indices (body length and width) of *Maritrema gratiosum* distomes in 20% chicken serum (+ 100 U/mL penicillin + 100 μg/mL streptomycin) and 40% chicken serum (+ 200 U/mL penicillin + 200 μg/mL streptomycin) over a 240-h period post-excystment at 40 °C

Treatment group	20% CS group		40% CS group	
Growth Indices	Range	Mean ± S.E	Range	Mean ± S.E
Body length (µm)	473.76–1324.55	853.71 ± 23.44	566.68–1632.14	997.58 ± 32.72
body width (µm)	183.46–412.97	307.45 ± 7.07	175.11–427.82	315.65 ± 7.63

Due to the experimental design, there was a high percentage of censored data (51.7% and 50% for groups 1 and 2, respectively), representing specimens that left the test rather than died (see Sect. "Assessing the performance of the *in vitro* culture method"). Survival curves generated by the Kaplan–Meier method (Stalpers and Kaplan 2018) did not show abrupt declines throughout the experiment. Kaplan–Meier survival curves are presented as a standard format for survival analysis data, facilitating comparisons with other research studies.

#### Growth indices

**Fig. 3:**
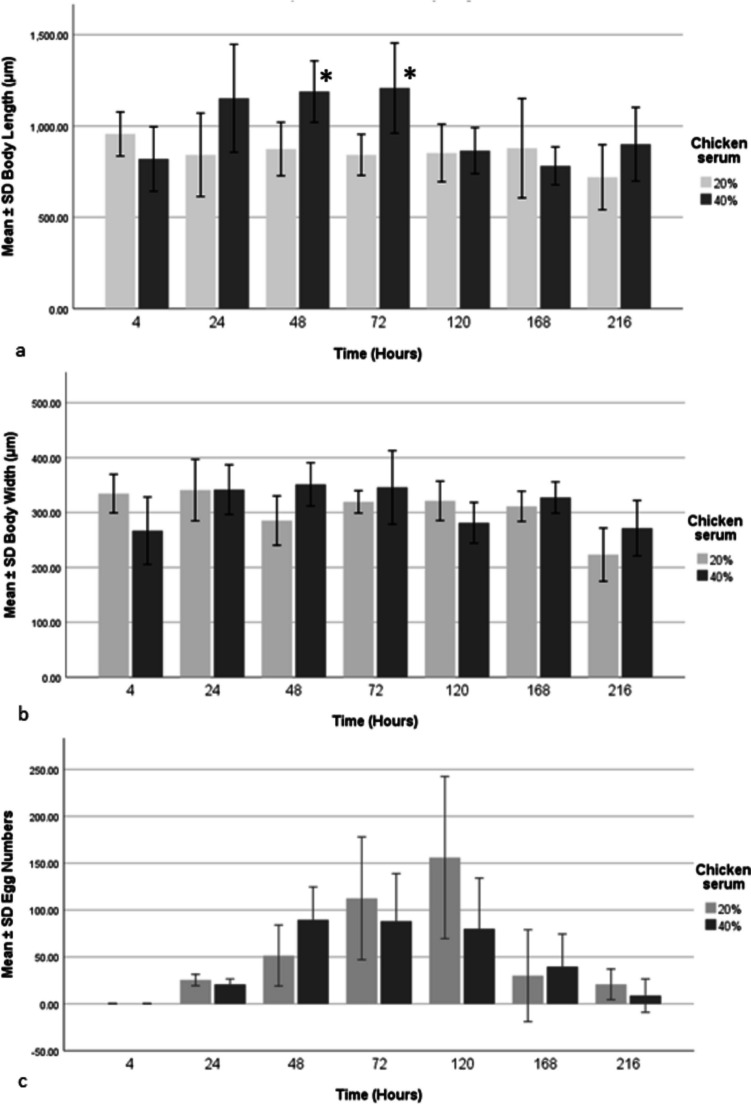
Comparison *Maritrema gratiosum* distome length (**A**), width (**B**), and egg numbers (**C**) at seven time points over 216 h post excystment cultured in NCTC 109 with either 20% (+ 100 U ml^-1^ penicillin + streptomycin 100 μg mL^-1^) or 40% chicken serum (+ 200 U ml^-1^ penicillin + streptomycin 200 μg mL^-1^) at 40ºC. *Statistically significant differences (*p* < 0.007) were found in body length. Body length and width were compared using independent sample t-tests with a Bonferroni correction (adjusted significance *p* < 0.007) to account for multiple comparisons. The figures were produced using IBM SPSS Statistics version 25

The growth indices for the two treatment groups are summarized in Table 2. Body length and width showed no consistent trend over time. However, body lengths in the 40% CS group were significantly larger than those in the 20% group at 48 and 72 h (independent sample T test, *p* < 0.001, Fig. 3A). Body length in the 40% group was 1.36 times greater than that in the 20% group at 48 h and 1.43 times greater at 72 h. Body width did not differ significantly between groups at any time point (Fig. 3B).

Egg numbers ranged from 0 to 269 (mean: 57.22 ± 9.18 SE) in the 20% CS group and 0 to 428 (mean: 89.09 ± 15.90 SE) in the 40% group. A Mann–Whitney U test, following a significant normality test, showed no significant differences in egg numbers between groups at any time point (Fig. 3C). Eggs were observed from the second day, peaking at the fifth day in the 20% group and the second day in the 40% group, then decreasing towards the end of the experiment.

### Developmental morphology and structural observation by laser scanning confocal microscopy (LSCM)

**Fig. 4:**
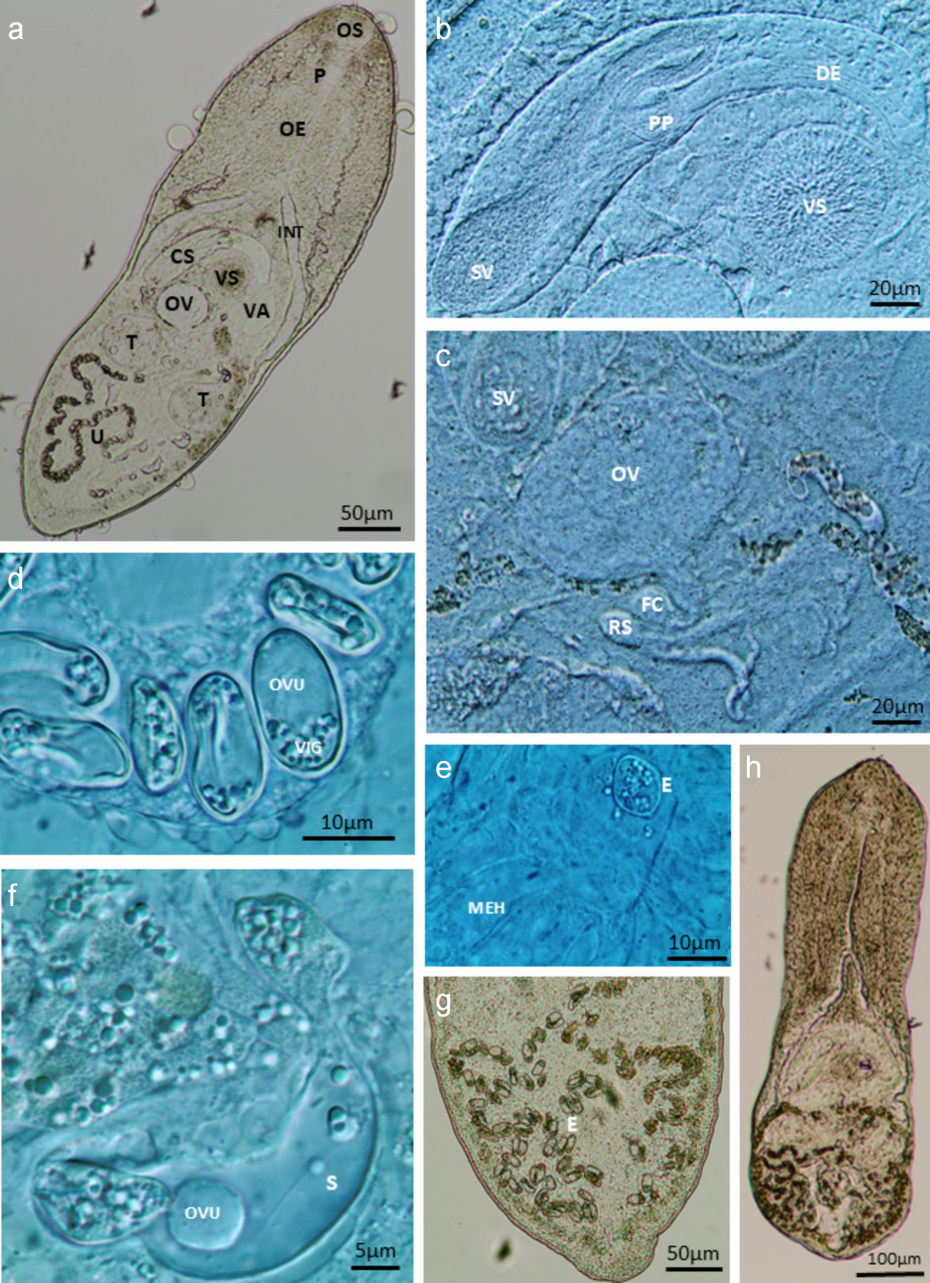
**A** Whole worm micrograph, note that the distal uterus (U) is full of eggs (NCTC 109 supplemented with 40% CS and antibiotics for 48 h, ventral view). **B** Higher magnification showing the male copulatory organs (NCTC 109 supplemented with 20% CS and antibiotics for 24 h). **C** Higher magnification showing the female copulatory organs (NCTC 109 supplemented with 40% CS and antibiotics for 72 h). **D** Eggs in the uterus; note the cells at one or both ends of the egg, which are likely vitelline globules, and an ovum in the middle. **E** *Receptaculum seminis* and a developing egg inside; note the eggshell and visible Mehlis gland (NCTC 109 supplemented with 20% CS and antibiotics for 24 h). **F** Higher magnification of the uterus loop; note an ovum and a spermatozoan inside (NCTC 109 supplemented with 20% CS and antibiotics for 72 h). **G** Lower magnification of the uterus loop; note both normal and abnormal eggs (NCTC 109 supplemented with 20% CS and antibiotics for 168 h). The abnormal eggs looked granular without shells or had deformed shells and had accumulated in the distal part of the uterus. **H** An adult cultured in NCTC 109 supplemented with 20% CS and antibiotics for 216 h, showing signs of senescence. Whole worm ventral view. **CS**: cirrus sac. **DE**: *ductus ejaculatoris*. **E**: egg. **FC**: fertilization chamber. **INT**: intestine caecum. **MEH**: Mehlis gland. **OS**: oral sucker. **OV**: ovary. **OVU**: ovum. **P**: pharynx, **PP**: *pars prostatica*. **RS**: *receptaculum seminis*, **S**: spermatozoan, **SV**: seminal vesicle. **T**: testis. **U**: uterus. **VA**: vagina. **VIG**: vitelline globules, **VS:** ventral sucker. Figures produced by Olympus cellSens software

At 4 h, all body organs of the young adults were visible, but no eggs were present in the uterus. By 24 h, a few eggs began appearing from the proximal uterus, and egg formation was observed in the fertilization chamber (Fig. 4E). These eggs were small, oval, and had thin shells. At 48 h, typical *M. gratiosum* morphology was evident, the distal uterus was full of eggs (Fig. 4A), with no significant changes in the male and female copulatory organs over the 10-day observation period (Figs. 4B, C). The 40% CS group showed larger body lengths compared to the 20% group at 48 and 72 h (Fig. 3A), but no other significant morphological differences were observed between the treatment groups.

By 48 h, numerous spermatozoa were present in the cirrus, *ductus ejaculatorius*, and vagina. More eggs appeared, these eggs contain at one or both ends of the egg, which are likely vitelline globules, and a transparent ovum in the middle (Fig. 4D). At 72 h, additional eggs were found in the uterus, along with developing eggs and spermatozoa (Fig. 4F). These eggs were filled with vitelline cells or globules and dividing embryos. By 120 to 168 h, the uterus was full of eggs. Abnormal eggs looked granular without shells or had deformed shells, were numerous in the proximal uterus, while normal eggs, which displayed shells, vitelline cells or globules, and ova, were more abundant in the distal uterus (Fig. 4G). At 216 h, the flukes showing signs of senescence, appeared shrunken, less active, and had wrinkled teguments (Fig. 4H). Most of the uterus contained abnormal eggs and cell debris, and opercula on eggs were not confirmed.

**Fig. 5:**
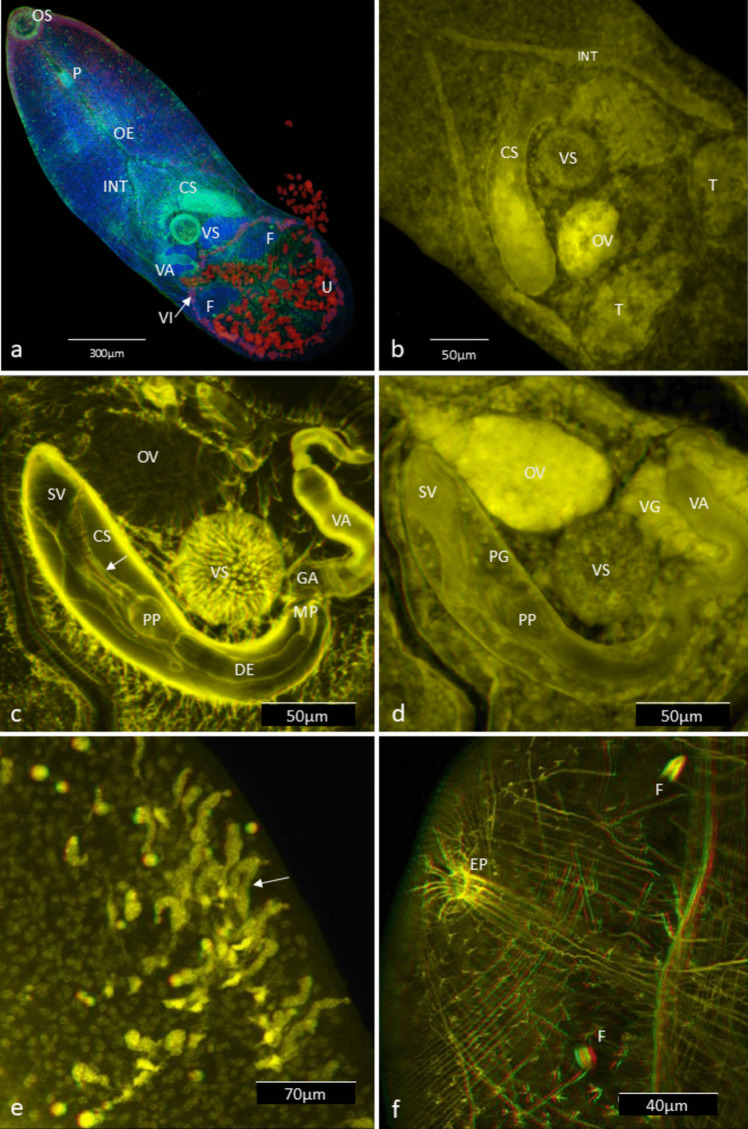
*Maritrema gratiosum*-morphology under LSCM. **A** DAPI (blue) and phalloidin (green) stain combined white light images (red) (NCTC-109 supplemented with 40% chicken serum and antibiotics for 120 h, dorsal view). Note that the imaged uterus was full of eggs (red), some of which have escaped the uterus due to a fracture in the specimen. **B** Reproductive organs stained with DAPI (NCTC-109 supplemented with 20% CS and antibiotics for 48 h, ventral view). **C** Male reproductive system (NCTC-109 supplemented with 40% CS and antibiotics for 120 h, ventral view of anaglyph 3D image). Note the ligament connecting the *pars prostatica* and seminal vesicle (arrow), showing phalloidin channel only. **D**. The same specimen as C but showing DAPI channel only. **E** Tegumental structures showing DAPI channel only (NCTC-109 supplemented with 20% CS and antibiotics for 48 h). Note the “finger-like” structures (arrow) near the surface of the worm. **F** Excretory pore showing clear radial and circular muscles and two nearby flame cells (NCTC-109 supplemented with 40% CS and antibiotics for 120 h and stained with phalloidin). **CS**: cirrus sac. **DE**: *ductus ejaculatoris*. **EP**: excretory pore. **F**: flame cells. **GA**: genital atrium. **INT**: intestine caecum. **MP**: male pore at genital atrium. **OE**: oesophagus. **OS**: oral sucker. **OV**: ovary. **PG**: prostate glands. **P**: pharynx. **PP**: *pars prostatica*. **SV**: seminal vesicle. **T**: testis. **U**: uterus. **VA**: vagina. **VG**: vaginal gland. **VI**: vitellaria. **VS**: ventral sucker. Figures produced by the embedded software of confocal microscopy (Leica TCS 2 AOBS LSCM)

Confocal microscopy provided insights into the musculature and organ structures. Phalloidin staining revealed typical digenean muscle patterns, including circular, longitudinal, and diagonal muscles, with denser bands in the forebody compared to the hind body (Fig. 5A, F). The copulatory organs were well-developed, with muscular structures in the cirrus sac, seminal vesicle, and *pars prostatica* (Fig. 5C). A previously unreported ligament was found connecting the *pars prostatica* and seminal vesicle. The *pars prostatica* was ampulla-shaped and divided into several chambers (Fig. 5D). Apparent glandular “finger-like” structures in the tegument were observed in the forebody (Fig. 5E). These structures were characterized by sinuous ducts and moderate fluorescence in the DAPI channel. However, the cell bodies of these glands were not visible, possibly because they fused with the secretions. Under light microscopy, these glandular structures were eosinophilic and appeared refractive in the present study (data not shown).

**Fig. 6:**
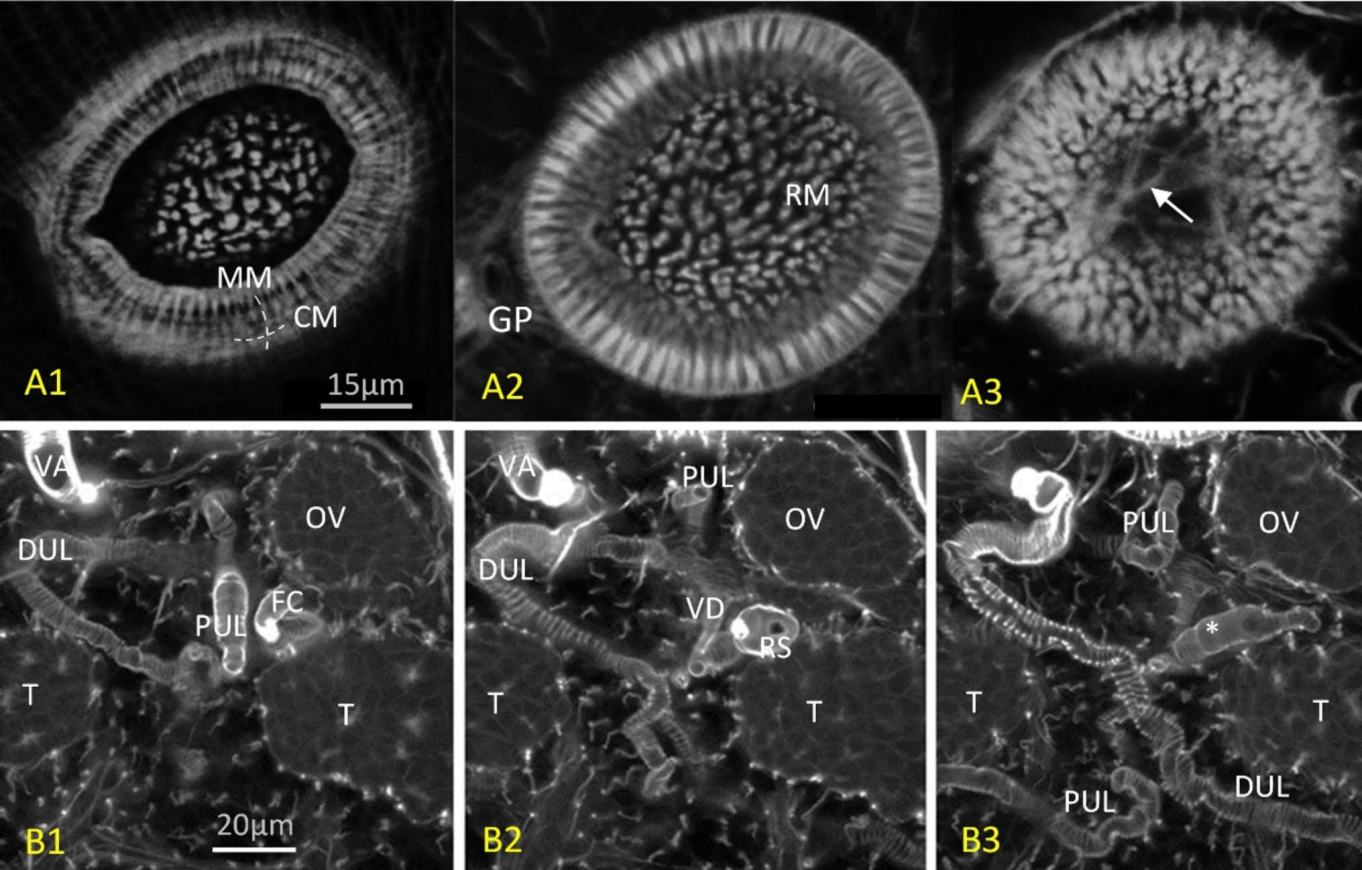
**A** Different optical sectioning depths of the ventral sucker of a newly excysted specimen of *Maritrema gratiosum* that was subsequently stained with × 8 phalloidin. A1. The exterior-most circular and meridial muscles. A2. The radial muscles inside and the genital pore on the side. A3. Extra supportive muscles (arrow) at the base of ventral sucker. **B** Ootypic junction of a newly excysted *M. gratiosum* stained with × 8 phalloidin moving from the dorsal side (left) to the ventral side (right). B1. Image showing ovary, fertilisation chamber, proximal uterus loop, distal uterus loop, testis, and vagina. B2. Image showing PUL and DUL, *receptaculum seminis*, ovary, testes, vagina, and vitelline duct. B3. The duct (asterisk) connecting the fertilisation chamber (can only be seen in B2) and (can only be seen in B1). Figures produced by the embedded software of confocal microscopy (Leica TCS 2 AOBS LSCM). **CM**: circular muscle. **DUL**: distal uterus loop. **FC**: fertilisation chamber. **GP**: genital pore. **MM**: meridial muscles. **OV**: ovary. **PUL**: proximal uterus loop. **RM**: radial muscle. **RS:** *receptaculum seminis. T*: testis. **VA**: vagina. **VD**: vitelline duct

**Fig. 7:**
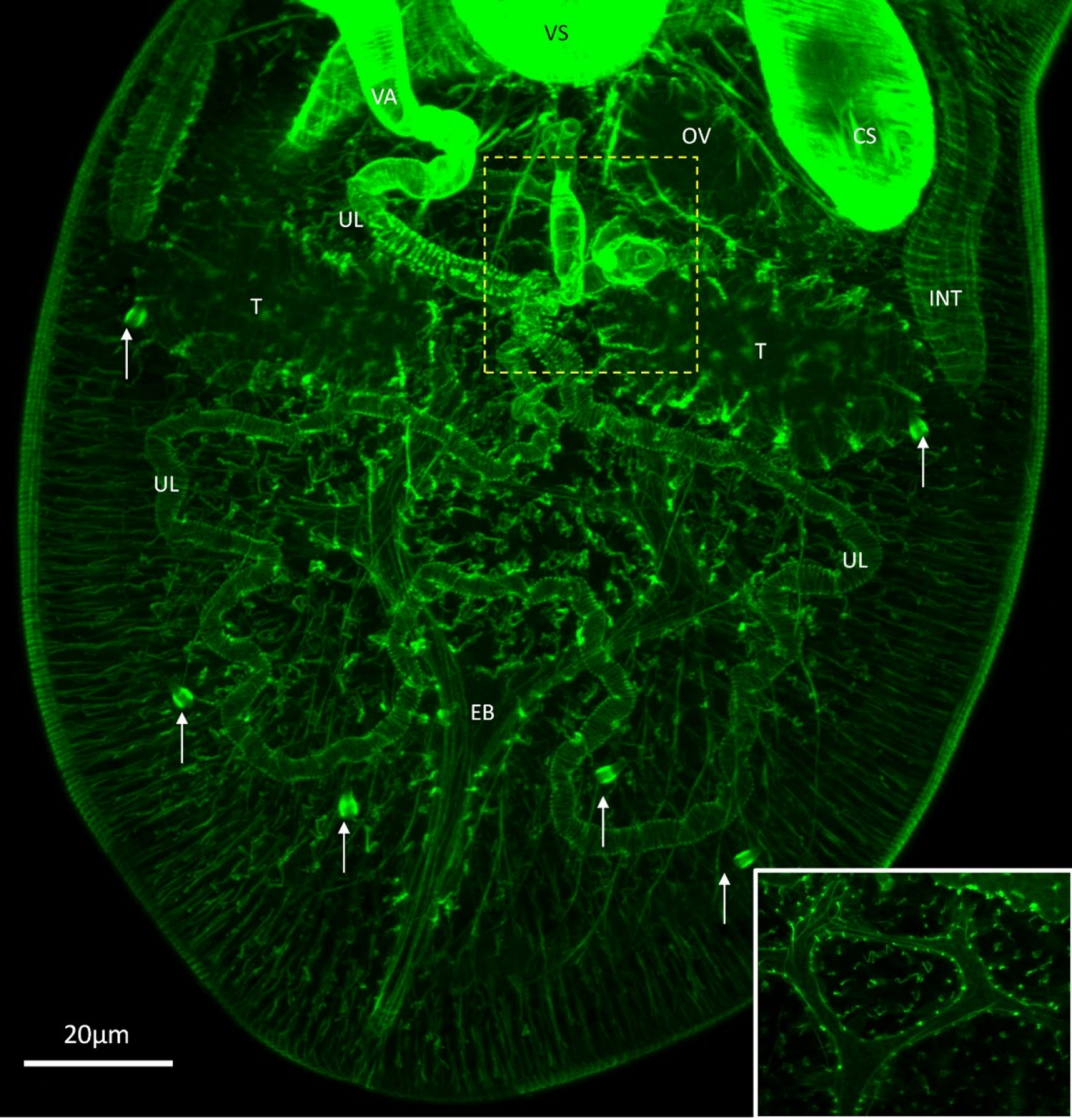
Newly excysted specimen of *Maritrema gratiosum* stained with × 8 phalloidin. Focusing on internal organs of the hind body of a newly excysted *M. gratiosum* stained with × 8 phalloidin. Dorsal view. The cirrus sac, excretory bladder, intestine caecum, ootypic junction (yellow square), ovary, vagina, ventral sucker, uterus loops and six flame cells (arrows) were clearly visible. Inset figure: Excretory bladder; note the racket-shaped excretory bladder with four side branches. Figures produced by the embedded software of confocal microscopy (Leica TCS 2 AOBS LSCM). **CS**: cirrus sac. **EB**: excretory bladder. **INT**: intestine caecum. **OV**: ovary. **VA**: vagina. **VS**: ventral sucker. **UL**: uterus loops

The ootypic junction included the ovary connected to a short oviduct, leading to a fertilization chamber and a *receptaculum seminis*. Laurer’s canal was not observed, and the configuration was similar to earlier descriptions (Fig. 6B), though further imaging is needed for confirmation. The excretory system displayed a racket-shaped bladder with two branches, differing from the typical Y-shaped or V-shaped bladders in Microphallidae (Fig. 7). The exact number of flame cells could not be determined.

## Discussion and conclusions

The development of long-term *in vitro* culture techniques for digeneans reduces reliance on vertebrate animals and offers tools for anthelmintic efficacy testing (Pung et al. 2009). Such techniques provide stable sources of target life stages crucial for drug screening and pharmacological studies, potentially reducing animal use. *In vitro* culture has been used for efficacy testing of single chemicals like piplartine on *S. mansoni* schistosomula (De Moraes et al. 2012), medium-throughput phenotypic screening (Abdulla et al. 2009), and high-throughput drug screening (Tavares et al. 2016). It has also been applied to liver flukes, such as plumbagin targeting *F. hepatica* (Lorsuwannarat et al., 2014) and plant extracts on *Fasciola gigantica* Cobbold, 1855 (see Vera-Montenegro et al. 2008), and recently to RNA interference studies in liver flukes (McCusker et al. 2020).

*In vitro*-produced flukes may exhibit different morphology and biology from those produced *in vivo*, with mortality and phenotypic changes being key evaluation parameters. Assessing viability and egg production is crucial for understanding basic biology (Stephenson 1947; Fujino et al. 1977; Davies and Smyth 1979; Pung et al. 2009, 2011; Tavares et al. 2016). This study investigates the impact of serum on the *in vitro* survival and egg production of newly excysted *M. gratiosum* metacercariae from *S. balanoides*. Light microscopy provided detailed morphological data, while LSCM offered insights into musculature development.

The *in vitro* culture requirements vary between progenetic and non-progenetic species and among species within the same genus. This study used media previously employed in microphallid culture, including PBS, EMEM, and NCTC109, yielding comparable results to earlier research. Previous attempts to culture *M. gratiosum* and other microphallids aimed to produce ovigerous adults with normal eggs but often fell short due to shorter survival and abnormal egg presence (Fujino et al. 1977; Davies and Smyth 1979; Zaben 1988; Fredensborg and Poulin 2005; Pung et al. 2009; 2011; West et al. 2014). This study achieved superior image quality, observed early spermatogenesis, validated self-fertilization, and included rigorous statistical analyses of survival, body dimensions, and egg numbers. It also provided valuable information for musculature research using LSCM and discussed key aspects such as excystment percentage, culture media optimization, egg development, and comparative anatomy for microphallids.

### Excystment percentage

In this study, *M. gratiosum* metacercariae were successfully excysted by incubating them in 0.01 M PBS at 40 °C for 2 h, achieving nearly 100% excystment. This temperature was selected based on the body temperature of avian final hosts. The excystment rate was similar with NCTC-109, but slower than the 15-min excystment observed by Irwin (1983) using Hanks Balanced Salt Solution (HBSS) with trypsin and bile salt, which achieved an 80% success rate. Irwin did not specify the excystment of the remaining 20%. Zaben (1988) reported a slower excystment rate in 40% seawater compared to the 86.7% rate within 12 h in this study. Although Irwin (1983) noted high efficiency with his solution (87.5% in 2 h), the excysted metacercariae had high mortality and inactivity. In *Maritrema novaezealandense* Martorelli, Fredensborg, Mouritsen et Poulin, 2004, metacercariae excysted within 4 h in 0.85% NaCl or NCTC-109 with 20% or 40% serum, with lower rates in 0.85% NaCl (Fredensborg and Poulin 2005). For *Microphallus japonicus* Osborn, 1919, metacercariae excysted faster in 0.85% NaCl than in Krebs–Ringer’s solution, with temperatures from 33–41 °C accelerating the process; over 80% excysted within an hour at 37 °C (Fujino et al. 1977).

The ease of excystment for *M. gratiosum* suggests it is an intrinsic process triggered by elevated temperature. Similarly, *Microphallus opacus* (Ward, 1894) Ward, 1901, metacercariae can excyst in 0.6% saline, amphibian Ringer’s solution, distilled water, or conditioned tap water at room temperature (Caveny and Etges 1971). Irwin (1983) used transmission electron microscopy (TEM) to study structural changes in *M. gratiosum* metacercariae during excystment, noting that the cyst wall, comprised of four layers, underwent changes, with less acid phosphatase activity in the glycocalyx post-excystment. This suggested that excystment involves initial metacercarial activity softening the cyst wall, with enzymes and chemicals in the excystment fluid aiding this process.

In contrast, *M. abortivus* required a more complex excystment fluid: a bicarbonate saline solution with sodium taurocholate and trypsin, combined with a 0.02 M HCl solution containing L-cysteine (Irwin et al. 1984). This mixture achieved a 95% excystment rate within 15 min at 40 °C (Saville and Irwin 1991). The relative ease of excystment in *M. gratiosum* may contribute to its low host specificity among microphallid species.

### The impact of culture medium conditions on *in vitro* culture performance

The choice of NCTC-109 for culturing *M. gratiosum* was influenced by its successful use for *M. novaezealandense in vitro* (Fredensborg and Poulin 2005). Although Zaben (1988) media options, such as medium 199 and NCTC 135, were not tested due to unawareness at the time, nutrient supplements like FBS and CS were evaluated. NCTC-109, developed by the National Cancer Institute for mouse cell lines, was used successfully in this study as well as for other microphallids (Fujino et al. 1977; Fredensborg and Poulin 2005).

The marine environment in which *M. gratiosum* lives may influence the choice of culture medium. In an *in vitro* culture study on marine digenean parthenitae, an osmolality of approximately 900 mOsm and 4% penicillin–streptomycin–neomycin was found to optimise survival for three different marine digeneans. Low osmolality levels, closer to freshwater conditions, led to early death of rediae (Lloyd and Poulin 2011).

The body length of the flukes initially increased but later decreased, while body width remained consistent. Egg counts peaked at different times depending on the serum used, with FBS (in the pilot studies, data not shown) and CS showing peak counts at 70 or 93 h and 48 h, respectively. Chicken serum was preferred due to the avian final hosts of *M. gratiosum*. Early studies on *F. hepatica* highlighted the necessity of an energy supply for *in vitro* cultures, and various nutrient sources were tested. While some, like glucose and fructose, were beneficial, others, like embryo extracts, were inhibitory (Davies and Smyth 1979; Rohrbacher 1957).

The maximum lifespan of *M. gratiosum* in nature is unknown, but *in vitro* cultures might extend survival with optimized conditions. Comparisons to other studies showed that *M. similis* and *M. opacus* could survive up to 30 days and 4 weeks, respectively, in culture. Although the 40% CS group showed potential superiority, factors like antibiotic concentration might have influenced results (Ractliffe et al. 1969; Moné et al. 2010). The effect of antibiotics on survival was not fully established but was consistent with similar studies.

Assessing viability through movement is standard, but more sophisticated methods like fluorimetry and impedance-based assays exist (Rinaldi et al. 2015; Tavares et al. 2016). In the present study, movement was a key indicator, though it was noted that flukes might appear dead due to a static period but recover with stimulation. Abnormalities observed in flukes cultured in PBS or EMEM were observed, suggesting possible malnutrition and shorter lifespan under current conditions. Overall, the body size of cultured flukes resembled those from the wild (Hadley and Castle 1940), indicating that the current protocol supports growth similar to natural conditions.

### Egg development

In the study of egg production for the digenean fluke *M. gratiosum in vitro,* several key observations and comparisons with previous research were noted. Egg production began at 24 h and peaked around the third to fourth day before decreasing. This pattern is consistent with earlier studies, such as Zaben (1988), which also observed a reduction in egg production after a few days.

The current study found a maximum mean egg number of 156 eggs from nine flukes at 120 h in the NCTC 109 plus 20% FBS group (data not shown). In contrast, Zaben reported a maximum of 54.94 eggs from 16 flukes at 72 h using medium 199 plus 10% FBS. Additionally, this study observed that peak egg production in the 20% CS group was delayed compared to the 40% CS group.

Comparing these results with other studies, such as Fredensborg and Poulin (2005) and Fujino et al. (1977), reveals that serum supplementation generally improves egg production. However, despite the beneficial effects of serum, *in vitro* egg production was still lower than *in vivo* production. For example, *in vivo* production often yielded better results in terms of quantity and timing, as seen in studies on *M. japonicus* (see Fujino et al., 1977 ) and *M. similis* (see Davies and Smyth, 1979).

The addition of chicken serum in the current study enhanced egg production, with no significant difference between 20 and 40% serum concentrations. However, higher serum concentration was associated with an earlier peak in egg production and higher mean egg number. This finding aligns with the observations from Pung et al. (2009) and Fujino et al. (1977), which also reported improved egg production with serum supplementation.

Overall, while serum supplementation supports the general health and egg production of microphallids *in vitro*, it does not fully replicate the conditions necessary for optimal egg production found *in vivo*. This underscores the complexities of *in vitro* culturing and the challenges of achieving *in vivo*-like conditions.

### Confocal microscopy observation of musculature and internal organs

#### Body wall musculature

The body wall musculature of *M. gratiosum* in this study includes outer circular, middle longitudinal, and inner diagonal muscle fibres, a pattern consistent with that of other platyhelminths. This arrangement is conserved across various digenean species, such as *Microphallus piriformes* Galaktionov, 1983, *M. pygmaeus*, *Levinseniella brachysoma* (Creplin, 1837) Stiles et Hassall, 1902 (see Krupenko and Dobrovolski 2018)*, Echinostoma caproni* Richard, 1964 (see Šebelová et al. 2004), *S. mansoni*, (see Mair et al*.*, 2000), and even the free-living tubellarian *Macrostomum hystricinum marinum* Rieger, 1977 (see Rieger et al. 1994). These muscles play crucial roles in locomotion and attachment. Specifically, the longitudinal and circular muscles work to stretch and shorten the body, while the diagonal muscles enable side-to-side movement and body torsion. The dorso-ventral muscles are essential in maintaining the flattened body shape typical of flatworms, with their absence resulting in a barrel-shaped body, as seen in *M. hystricinum marinum*.

Somatic muscles, together with suckers and tegumental spines, are integral for attachment. In microphallids, surface spines aid in locomotion and attachment. The force exerted by these spines is driven by the body wall musculature, which works through the ventro-dorsal muscles and the ventral concavity—a term used to describe the compact muscular wall found in species lacking a ventral sucker. This structure acts similarly to a sucker and generates negative pressure through muscle contractions.

In addition to the general body musculature, the musculature of the suckers in digeneans, including *M. gratiosum*, features surface circular and meridial (longitudinal) muscles and inner radial muscles. Meridial muscles open the sucker, radial muscles close it into a cup shape, and circular muscles create the suction force. The presence of web-like protractor muscles at the base of the ventral sucker and symmetrical ventro-dorsal muscles in the oral sucker further supports the functionality of these structures, enhancing attachment and feeding efficiency. These findings align with the supportive structures described by other researchers (Krupenko and Dobrovolski 2015).

#### Reproductive organs

*Maritrema gratiosum* is progenetic, similar to other species in the Microphallidae family, exhibiting well-developed reproductive organs in newly excysted young adults, with no further development of these organs or somatic musculature over a 5-day observation period. In contrast, non-progenetic species like *E. caproni* show significant morphogenesis of reproductive organs in pre-ovigerous adults, while somatic muscles remain relatively unchanged.

The study did not observe the “ootype” or Mehlis gland, although Hadley and Castle (1940) described the ootype as the “*receptaculum seminis*” and identified a “shell gland” surrounding it. This shell gland may correspond to the Mehlis gland. Ova were seen rotating in the fertilization chamber of living specimens, but the precise timing and location of shell formation remain unclear. The shell gland was not visible even under the DAPI channel, which highlights tissue cell nuclei. Sphincters at the “ootypic junction” suggest a specific sequence for egg formation, indicating a need for more research to clarify egg formation in *M. gratiosum*.

Differences between the current study and Hadley and Castle (1940) original description could be attributed to variations in microscopy techniques (confocal vs. light microscopy) and specimen compression. Future studies employing LSCM with sequential specimens, along with neurotransmitter identification in reproductive organs, might provide deeper insights into the fertilization process and sequential changes in reproductive organs.

#### Glandular structures in the tegument

In the present study, apparent glandular structures in the tegument of cultured *M. gratiosum* adults were observed using confocal microscopy. These structures, previously described in microphallids such as *Maritrema* species (Ching, 1963; Smith, 1983; Benjamin and James, 1987; Galaktionov, 1989; Tkach, 1998) and *M. similis* (see Davies, 1979) using light microscopy, were found to be located in the forebody. Transmission electron microscopy studies have documented various types of tegumental glands in microphallids. For instance, spherical granules in cells beneath the tegumental syncytium were observed in newly penetrated *M. gratiosum*, with changes in cell types and granule contents noted during development. Forebody gland cells, different from tegumental glands, have been described with thicker ducts and electron-dense granules. These glands were initially identified in *M. similis* as having a nucleated cell body with electron-dense granules and were involved in secreting complex materials like diastase-resistant neutral mucosubstances and cholinesterase, which might help the fluke evade host immune responses. Other studies, including those by Benjamin and James (1987) and Galaktionov (1996), have described various gland cells in the tegument, including mucopolysaccharide gland cells and cystogenous gland cells, which contribute to cyst formation and possibly resist host immune systems.

In the current study, the “finger-like” structures observed in the tegument under LSCM appear similar to previously described forebody glands. The use of DAPI in this study is novel for microphallids, as it primarily stains DNA and RNA but can also detect other autofluorescent materials like chitin (Zirbel et al. 2007). Further investigation is needed to confirm the function of these glands and ducts in *M. gratiosum*. The study highlights the potential for DAPI to reveal internal structures in microphallids, but additional evidence is required to fully understand the role of these observed glandular structures.

## Final remarks and conclusions

The present study offers an in-depth examination of the developmental morphology of *M. gratiosum*, the type species for the genus *Maritrema*, from excystment to ovigerous adults. This research marks the second *in vitro* culture study of *M. gratiosum* in 37 years, following the work of Zaben (1988). The study revealed that *M. gratiosum* has a longevity of 10 days under the current conditions, though this duration should be confirmed through future research. The lifespan of adult digeneans can have significant ecological implications, particularly for species like *M. gratiosum* that are transmitted by migratory birds. Extending the longevity of cultured flukes through improved culture conditions—such as media composition, food sources, and gas phase—could enhance their development and oviposition. Future advancements might include using novel techniques like xWORM (Rinaldi et al. 2015) to assess the health and vitality of cultured adults, as well as employing environmental DNA methods to detect the presence of eggs in natural settings, validating oviposition in the wild.

The study confirmed the precocious sexual development and self-fertilization ability of *M. gratiosum*, highlighting the need for further exploration of its progenesis and its evolutionary and ecological implications. Using LSCM, the research provided the first detailed description of the musculature of cultured *M. gratiosum* at various developmental stages. While the muscular system observed aligns with those of other microphallids, this study identified previously unobserved structures, such as a ligament connecting the pars prostatica and seminal vesicle and a racket-shaped excretory bladder with two branches on each side. These findings underscore the potential of modern microscopy techniques to advance the taxonomy of digeneans based on their morphology.

The insights gained from this study contribute to a better understanding of the neuro-muscular coordination in *M. gratiosum*, offering opportunities for future research into its excretory and reproductive biology. Although the impact of *M. gratiosum* on wading birds remains unclear, the increasing stress on seabird populations from environmental changes could amplify the effects of parasitic infections. This study enhances our knowledge of microphallids and provides new tools and insights for investigating host-parasite interactions.

## Data Availability

Research data is available on request by contacting the lead author.

## Declarations

### Research data

Available on request by contacting the lead author.

### Clinical trial

Clinical trial number: not applicable.
